# Alzheimer’s Disease Microbiome Is Associated with Dysregulation of the Anti-Inflammatory P-Glycoprotein Pathway

**DOI:** 10.1128/mBio.00632-19

**Published:** 2019-05-07

**Authors:** John P. Haran, Shakti K. Bhattarai, Sage E. Foley, Protiva Dutta, Doyle V. Ward, Vanni Bucci, Beth A. McCormick

**Affiliations:** a Department of Emergency Medicine, University of Massachusetts Medical School, Worcester, Massachusetts, USA; b Department of Microbiology and Physiological Systems, University of Massachusetts Medical School, Worcester, Massachusetts, USA; c Department of Bioengineering, Program in Engineering and Applied Sciences, University of Massachusetts Dartmouth, North Dartmouth, Massachusetts, USA; d Center for Microbiome Research, University of Massachusetts Medical School, Worcester, Massachusetts, USA; Yale School of Public Health; University of Chicago; Icahn School of Medicine at Mount Sinai

**Keywords:** Alzheimer’s Disease, dementia, elderly, gut-brain axis, intestinal homeostasis, intestinal microbiome

## Abstract

The microbiota-gut-brain axis is a bidirectional communication system that is poorly understood. Alzheimer’s disease (AD), the most common cause of dementia, has long been associated with bacterial infections and inflammation-causing immunosenescence. Recent studies examining the intestinal microbiota of AD patients revealed that their microbiome differs from that of subjects without dementia. In this work, we prospectively enrolled 108 nursing home elders and followed each for up to 5 months, collecting longitudinal stool samples from which we performed metagenomic sequencing and *in vitro* T84 intestinal epithelial cell functional assays for P-glycoprotein (P-gp) expression, a critical mediator of intestinal homeostasis. Our analysis identified clinical parameters as well as numerous microbial taxa and functional genes that act as predictors of AD dementia in comparison to elders without dementia or with other dementia types. We further demonstrate that stool samples from elders with AD can induce lower P-gp expression levels *in vitro* those samples from elders without dementia or with other dementia types. We also paired functional studies with machine learning approaches to identify bacterial species differentiating the microbiome of AD elders from that of elders without dementia, which in turn are accurate predictors of the loss of dysregulation of the P-gp pathway. We observed that the microbiome of AD elders shows a lower proportion and prevalence of bacteria with the potential to synthesize butyrate, as well as higher abundances of taxa that are known to cause proinflammatory states. Therefore, a potential nexus between the intestinal microbiome and AD is the modulation of intestinal homeostasis by increases in inflammatory, and decreases in anti-inflammatory, microbial metabolism.

## Abstract (precis)

Studies of the intestinal microbiome and AD have demonstrated associations with microbiome composition at the genus level among matched cohorts. We move this body of literature forward by more deeply investigating microbiome composition via metagenomics and by comparing AD patients against those without dementia and with other dementia types. We also exploit machine learning approaches that combine both metagenomic and clinical data. Finally, our functional studies using stool samples from elders demonstrate how the c microbiome of AD elders can affect intestinal health via dysregulation of the P-glycoprotein pathway. P-glycoprotein dysregulation contributes directly to inflammatory disorders of the intestine. Since AD has been long thought to be linked to chronic bacterial infections as a possible etiology, our findings therefore fill a gap in knowledge in the field of AD research by identifying a nexus between the microbiome, loss of intestinal homeostasis, and inflammation that may underlie this neurodegenerative disorder.

## INTRODUCTION

The concept of the “gut-brain axis,” which originated from behavioral studies in microbiome-reconstituted mice, has advanced current research supporting the concept that the microbiome may be responsible for some of the most devastating neurodegenerative disorders, including Alzheimer’s disease (AD) (1). This is further underscored by observations linking AD pathogenesis to chronic bacterial infections as a possible etiology (2, 3). Recent studies have investigated this connection, identifying significant changes in the proportion of certain microbiome taxa in AD patients (4, 5), and have correlated microbiota composition with levels of AD biomarkers in cerebrospinal fluid (4). Moreover, increased proportions of proinflammatory and reduced proportions of anti-inflammatory bacteria in the intestine are associated with systemic inflammatory states in patients with cognitive impairment and brain amyloidosis (6). Thus, a current tenet supports the idea that AD pathogenesis is not only closely related to the imbalance of the gut microbiome but may also originate in the gut (3, 7, 8).

The healthy human intestine involves a dynamic balance between the host immune response, the large population of resident bacteria, and the thin epithelial layer that separates them. Dysregulation of this balance can have serious consequences that may drive a variety of pathological conditions. The intestinal epithelia therefore serve as a physical barrier to microbial penetration and provide a sentinel system to warn immune cells of pathogen exposure or injury. This places them in an ideal position to regulate the balance between pro- and anti-inflammatory states. Previously, we characterized a balanced system at the intestinal mucosal surface in which eukaryotic ABC transporters and their efflux products play a fundamental role in immunomodulation (9, 10). This dynamic balance operates between homeostatic pathways that suppress immune responses to commensal bacteria (the P-glycoprotein [P-gp]/endocannabinoid axis) and inflammatory pathways that activate responses to pathogens or aberrant signals (multidrug-resistant protein 2 [MRP2]/hepoxilin A_3_) and can be unhinged by a dysbiotic microbiome (9). Dysregulation of this critical balance contributes directly to inflammatory disorders of the intestine. To understand more deeply how specific intestinal bacterial taxa associate with AD and the extent to which such taxa alter the balance of intestinal epithelial homeostasis, we explored the microbiome composition of nursing home (NH) elders with AD, no dementia, or other dementia types.

## RESULTS

### Elders with dementia have increased frailty and malnutrition scores.

One hundred eight NH elders were prospectively enrolled and followed for up to 5 months. Longitudinal stool samples, taken one time each month for a total of 300 samples, were collected. Of the 108 elders, 51 (47.2%) had no dementia, while 24 elders (22.2%) had AD and 33 elders (30.6%) had other dementia types. Of note, elders who were exposed to antimicrobials, had changes in medications, or required hospitalization during the study period were excluded from this study. A greater proportion of elders with AD or other dementia types were taking atypical antipsychotics and presented with higher malnutrition and frailty scores than did those with no dementia (Table 1). This is consistent with prior studies documenting that frailty and malnutrition are related to dementia (11–14). Strong associations have been shown among both frail and prefrail elders with a poorer cognitive status (15), and frailty has been linked to the level of AD pathology found on postmortem examination (12). Malnutrition is a common problem among NH elders, with upwards of 33% suffering from this condition (16). Among AD elders, the most malnourished display decreased cognitive and functional capacities (11).

**TABLE 1:** Clinical data by dementia type

Patient characteristic^*a*^	Data by dementia type			*P* value
	No dementia	Alzheimer’s disease	Other dementia	
Age (mean [SD]) (yr)	83.0 (10.2)	84.7 (8.1)	87.9 (7.9)	0.06
Age category (mean [SD])^*b*^	2.3 (1.0)	2.5 (0.8)	2.8 (0.8)	0.41
Male	8 (15.7)	4 (16.7)	6 (18.2)	0.96
Diabetic	11 (21.6)	5 (20.8)	9 (27.3)	0.80
Immunosuppressed	3 (5.9)	1 (4.2)	0 (0.0)	0.38
Malignancy	6 (11.8)	2 (8.3)	3 (9.1)	0.87
CKD	15 (29.4)	9 (49.5)	10 (30.3)	0.77
CCI score (mean [SD])	1.65 (1.7)	1.38 (1.2)	1.58 (1.6)	0.79
Medications				
PPI	16 (31.4)	1 (4.2)	6 (18.2)	0.024
Statin	11 (21.6)	4 (16.7)	8 (24.2)	0.79
Antipsychotic	0 (0.0)	4 (16.7)	2 (6.1)	0.013
Polypharmacy	34 (66.7)	14 (58.3)	22 (66.7)	0.75
Clinical scores				
Malnutrition (mean [SD])	1.7 (0.7)	2.3 (0.6)	2.3 (0.6)	<0.0001
Frailty (mean [SD])	2.9 (1.0)	3.4 (0.7)	3.7 (0.6)	0.003

a Data are presented as the number (%), unless otherwise specified. CKD, chronic kidney disease; CCI, Charlson comorbidity index.

b The age categories are as follows: category 1, 65 to 74 years; category 2, 75 to 84 years; category 3, 85 to 94 years; and category 4, ≥95 years.

We also noted a decreased prevalence of proton pump inhibitor (PPI) use among both dementia types. Besides atypical antipsychotics, other medications known to affect the microbiome did not differ among the three groups, including the proportion of elders with polypharmacy. Additionally, we did not note any significant difference in age, sex, or medical histories among these groups. The majority of elders with Alzheimer’s type dementia had moderate/severe symptoms defined by clinical dementia rating (CDR) scores of 2 to 3 (mean CDR score, 2.12; standard deviation [SD], 0.33), while elders with other dementia types had less severe symptoms (mean CDR score, 1.88; SD, 0.95; *P* = 0.026).

### Microbiome composition differs by dementia type.

**FIG 1:**
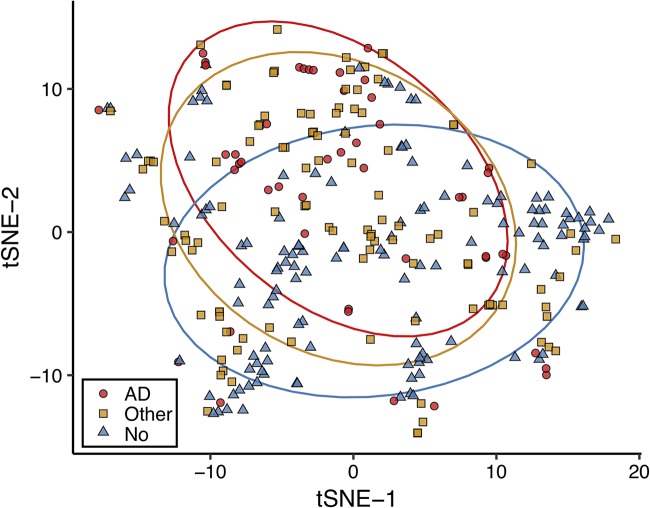
Microbiome diversity differs between Alzheimer’s disease elders and those with no dementia or other types of dementia. Stool samples from elders were sequenced via shotgun metagenomics. Samples were profiled for microbial species relative abundances by mapping reads to a NCBI bacterial genomes k-mer database with Kraken and by reconstructing the resulting relative abundance profile at the species levels with Bracken. Beta diversity was explored using Jaccard distances by *t*-distributed stochastic neighbor embedding (tSNE) for a measure of community species dissimilarity among samples collected from individuals without dementia (blue triangles), with Alzheimer’s disease (red circles), and with other dementia types (yellow squares). Each group is displayed with ellipses with a 95% confidence interval.

We evaluated the beta diversity of the intestinal microbiome between elders without dementia, with AD, and with other dementia types, using Jaccard distances as a measure of species-level community dissimilarity visualized using *t*-distributed stochastic neighbor embedding (tSNE) (Fig. 1). Elders with AD cluster away from those without dementia. Individuals with other dementia diagnoses are clustered with both cohorts. Moreover, Jaccard distances between samples from individuals with AD were more similar than those from individuals with no dementia or other dementia types (permutational multivariate analysis of variance [PERMANOVA], Jaccard distance *P* = 0.001).

**FIG 2:**
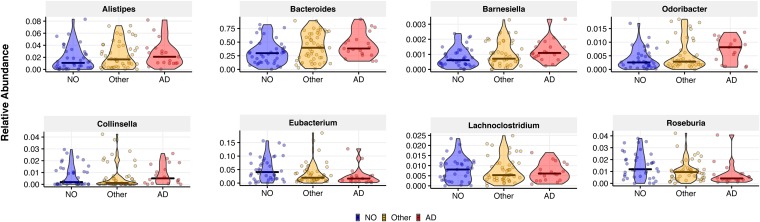
After adjusting for relative clinical covariates, microbiome composition differs at the genus level among elders with Alzheimer’s disease, no dementia, and other dementia types. We performed generalized mixed-effect modeling regression to predict genus-level proportions as a function of age, malnutrition, frailty, medications, and dementia state (no/other/AD). Patient ID was used as random effect to account for the repeated nature of our samples. Genera with greater than 0.1% mean relative abundance were significantly associated with Alzheimer’s disease (AD) or other dementia types (Other) in comparison to elders without dementia (NO). Only genera with a *P* value of <0.05 are presented, with relative abundance on the *y* axis.

Differences in the relative abundances of bacterial genera for elders with no dementia versus AD and other dementia types were characterized via generalized linear mixed models. Specifically, after using Kraken to map reads to a k-mer database of bacterial NCBI genomes (17) and estimating the relative abundances of each species with Bracken (18), we determined the relative abundances at the genus level. Beta-regression mixed-effect modeling was then used to predict the proportion of each genus as a function of the fixed effects of dementia status, age, malnutrition, frailty, and medications, including those we previously showed to significantly affect microbiome composition (19–21) (Table 1) and considering the individual elder as random effect. After adjusting for these clinical covariates, several genera were found to be significantly associated with AD (Fig. 2). When setting no dementia as the baseline, increased proportions of Bacteroides spp. (*P* = 0.031), Alistipes spp. (*P* < 0.001), Odoribacter spp. (*P* < 0.001), and Barnesiella spp. (*P* = 0.023) and decreased proportions of Lachnoclostridium spp. (*P* = 0.048) were present in AD elders, while increased proportions of Odoribacter spp. (*P* = 0.025) and Barnesiella spp. (*P* = 0.024) and decreased proportions of Eubacterium spp. (*P* < 0.001), Roseburia spp. (*P* = 0.034), Lachnoclostridium spp. (*P* = 0.048), and Collinsella spp. (*P* < 0.001) were seen in elders with other dementia types. Other studies, based on 16S rRNA sequencing, have found similar associations and also reported increases in *Bacteroides* spp. and *Alistipes* spp. (4), as well as decreased proportions of *Roseburia* spp. (5) in AD patients, consistent with what we report above. Differences in the other genera reported here are likely due to different methodologies applied, as we used shotgun metagenomics and mixed modeling rather than 16S rRNA and subject matching based only on age and sex. We have previously reported the importance of frailty and malnutrition on microbiome composition (19). There is also a growing awareness of medication effects on the intestinal microbiota (21, 22). We believe that including these key clinical variables is important when analyzing microbiome associations with disease outcomes.

### Accurate classification of AD individuals compared to those without dementia using metagenomic and clinical measures.

To identify taxonomic and clinical covariates that optimally differentiate AD elders from those with no dementia or other dementia types, two separate random forest classification algorithms were implemented (23, 24). The matrix of predictors included in each of the models consisted of species level, taxonomic relative abundances, and clinical measures. To account for the time dependence of our data, we performed 30 trials of the random forest classification algorithm, in which we randomly selected one sample per individual. A leave-one-out cross-validation approach was used, in which we trained the forest in *n* − 1 individuals and used the model to predict the membership of the left-out individual. The analysis was repeated 100 times using different random seeds. We averaged out-of-bag (OOB) error across the 30 × 100 trials and ranked predictors (taxa and clinical factors) with respect to their importance in classification according to their respective mean decreased accuracy distribution.

**FIG 3:**
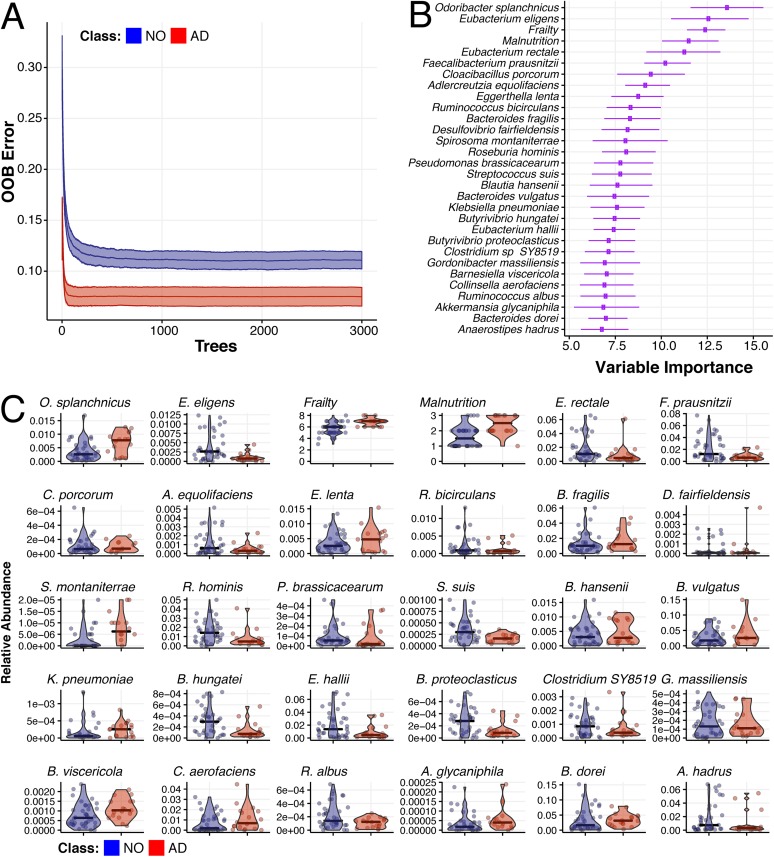
Microbiome species composition, combined with frailty and malnutrition, accurately classify individuals as having Alzheimer’s disease versus no dementia. Random forest classification was performed according to Alzheimer’s disease versus no dementia (AD versus NO) by selecting at random one sample per individual (30 trials per run), from 100 starting random seeds, and building 3,000 decision trees per trial. (A) Out-of-bag (OOB) prediction error as a function of the number of decision trees run for elders without dementia (NO, blue) or with Alzheimer’s disease (AD, red). (B) Ranking of forest predictors based on average variable importance (e.g., mean decreased accuracy) across the 30 × 100 random trials. The top 30 important features discriminating the AD and NO dementia are reported. (C) Relative abundances for each species selected or scoring number for frailty and malnutrition clinical variables are reported with mean (thick bar) and up to minimum and maximum values for elders without dementia (NO, blue) or with Alzheimer’s disease (AD, red). In panel B, species are ordered based on importance.

Our analysis identified two clinical parameters, increasing frailty and malnutrition, as predictors of AD dementia, as well as numerous microbial taxa with known association to inflammatory and neurological disorders (Fig. 3, and summarized in Table S1 in the supplemental material). Specifically, AD elders were characterized by lower proportions of key butyrate-producing species, such as members of the Butyrivibrio (B. hungatei and B. proteoclasticus) and Eubacterium (E. eligens, E. hallii, and E. rectale) genera, as well as Clostridium sp. strain SY8519, Roseburia hominis, and Faecalibacterium prausnitzii. Metagenomic analysis of metabolic pathways also indicates a similar pattern, in that elders without dementia have an increase in butyrate-coding genes from four separate butyrate biosynthetic pathways present in all bacteria in comparison to AD elders (Fig. S1).

10.1128/mBio.00632-19.1 FIG S1 Heat map of the relative abundance of each gene type in each individual and hierarchical clustering depicting butyrate biosynthetic gene pathways. We performed mixed-effect modeling on the butyrate genes to determine those significantly differentiating elders with Alzheimer’s disease (AD) versus those without dementia and to display them as heat map. Butyrate biosynthesis genes were grouped according to the four major butyrate biosynthetic pathways following the breakdown provided in reference 67. Download FIG S1, PDF file, 0.2 MB. Copyright © 2019 Haran et al. 2019 Haran et al. This content is distributed under the terms of the Creative Commons Attribution 4.0 International license.

10.1128/mBio.00632-19.3 TABLE S1 Bacterial species selected from variable importance plot of Alzheimer’s disease compared to elders with no dementia. We use the top 30 important features discriminating AD and no dementia from the forest predictors (Fig. 3) and placed them into 5 categories. Download Table S1, PDF file, 0.1 MB. Copyright © 2019 Haran et al. 2019 Haran et al. This content is distributed under the terms of the Creative Commons Attribution 4.0 International license.

Remarkably, AD elders had increased proportions of specific bacterial species that have associations with neurological disorders (including AD). These include Odoribacter splanchnicus, a bacterial species with genes that have been associated with the Alzheimer’s pathway (25). Other bacterial species identified as predictors of AD dementia include taxa known to cause inflammatory states, such as Bacteroides vulgatus (Fig. 3). This species has recently been identified as influencing neuroinflammatory signaling (26) and also has been associated with autism (27) and autoimmune diabetes (28, 29). AD elders were also depleted in Adlercreutzia equolifaciens, an equol-producing bacterium, which has beneficial effects in reducing experimental cutaneous inflammation in mice (30, 31) and the loss of which has been associated with the neurodegenerative disorder multiple sclerosis (32). Human pathogens Klebsiella pneumoniae, Bacteroides fragilis, and Eggerthella lenta were also shown to be increased in relative abundance in our AD elders (Fig. 3).

### Accurate classification of AD versus other dementia types using only metagenomic measures.

**FIG 4:**
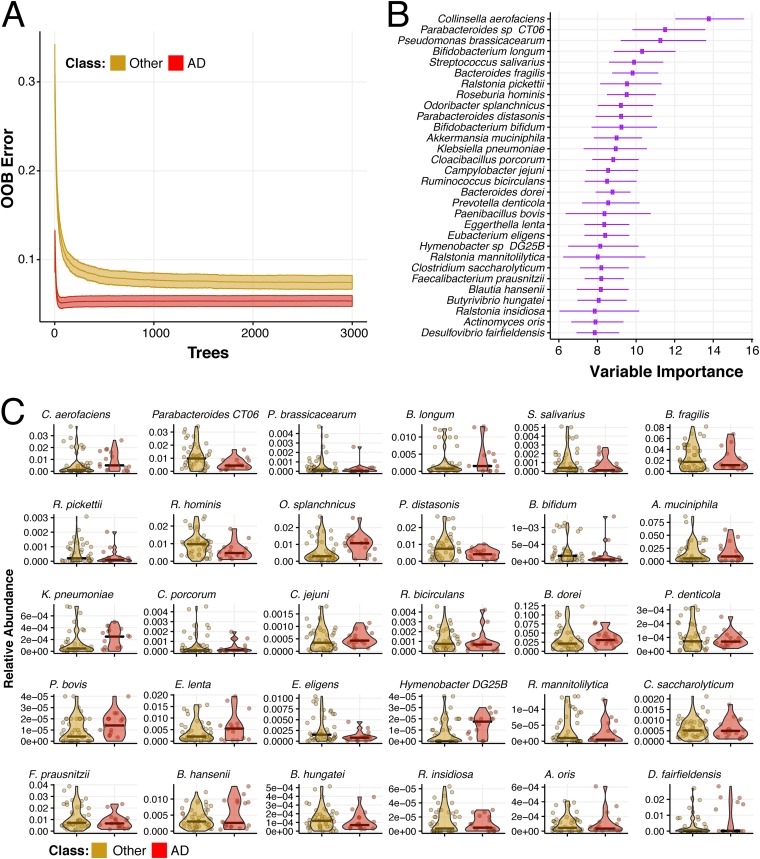
Microbiome species composition accurately classify individuals as having Alzheimer’s disease versus other dementia types. Random forest classification was also performed according to Alzheimer’s disease versus no other dementia diagnosis (AD versus other) by selecting at random one sample per individual (30 trials per run), from 100 starting random seeds, and building 3,000 decision trees per trial. (A) Out-of-bag (OOB) prediction error as a function of the number of decision trees run for elders without dementia (other, yellow) or with Alzheimer’s disease (AD, red). (B) Ranking of forest predictors based on average variable importance (e.g., mean decreased accuracy) across the 30 × 100 random trials. The top 30 important features discriminating AD and other dementia are reported. (C) Relative abundances for each species selected by the model are reported with mean (thick bar) and up to minimum and maximum values (dots) for elders with other dementia types (other, yellow) or with Alzheimer’s disease (AD, red). In panel B, species are ordered based on importance.

We applied random forest classification to discriminate AD elders from elders with other types of dementia (Fig. 4). Interestingly, no clinical variables emerged in the top 30 discriminating variables from this random forest analysis. Analogous to the AD versus no-dementia class comparison (Fig. 3), AD elders associated with enriched proportions of previously described dysbiotic bacteria, such as O. splanchnicus, E. lenta, and K. pneumoniae, and with decreased proportions of butyrate-producing B. hungatei, Blautia hansenii, E. eligens, R. hominis, Ruminococcus bicirculans, and F. prausnitzii (Fig. 4 and summarized in Table S2). Similarly, Bacteroides dorei, another bacterium with known association to autoimmune conditions and type 1 diabetes (29), is enriched in AD compared to both no-dementia and to other dementia type elders (Fig. 3C and 4C). Surprisingly, compared to elders with AD, elders with other dementia types had higher proportions of some opportunistic pathogens, including Ralstonia pickettii and Ralstonia mannitolilytica (33, 34), as well as B. fragilis, the most commonly isolated anaerobic pathogen (35). Our analysis also identified other species from genera that have been recently reported to have lower (Bifidobacterium bifidum) or higher (Prevotella denticola and Akkermansia muciniphila) proportions among AD patients (5).

10.1128/mBio.00632-19.4 TABLE S2 Bacterial species selected from variable importance plot of Alzheimer’s disease compared to elders with other dementia types. We use the top 30 important features discriminating AD and other dementia from the forest predictors (Fig. 4) and placed them into 5 categories. Download Table S2, PDF file, 0.1 MB. Copyright © 2019 Haran et al. 2019 Haran et al. This content is distributed under the terms of the Creative Commons Attribution 4.0 International license.

### Microbiome of AD elders modulates intestinal homeostasis through P-glycoprotein regulation.

**FIG 5:**
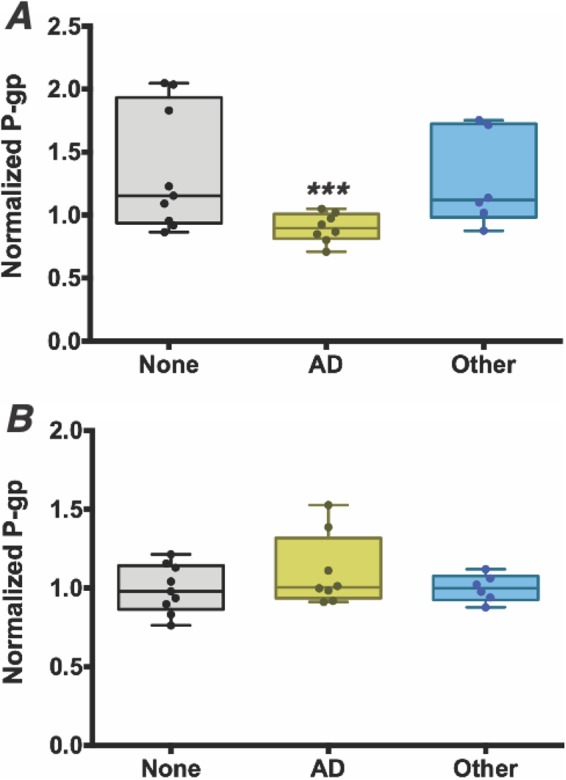
Intestinal microbiome of elders with Alzheimer’s disease induce significantly lower P-glycoprotein expression from intestinal epithelia cells, reflecting a higher level of inflammatory potential at the epithelial cell surface. (A and B) Supernatants were collected from stool samples from 9 randomly selected elders from each dementia classification group and incubated on T84 epithelial cells for 12 h prior to relative quantitation of P-gp by flow cytometry (A) or MRP2 by Western blotting (B). (A) P-glycoprotein expression was induced significantly less in samples from elders with Alzheimer’s disease (AD, yellow) than from those without dementia (none, gray) or with other dementia types (other, blue). Normalized P-glycoprotein (P-gp) expression is expressed on the *y* axis. (B) MRP2 did not show a significant difference in expression levels. ***, *P* < 0.05.

Our observed taxonomy associations in elders with AD may represent a proinflammatory microbiome that contributes to the inflammation-causing immunosenescence and is thought to be associated with the development of AD. To examine the notion that the microbiota from AD elders alone can differentially promote an inflammatory state, perhaps altering intestinal epithelial homeostasis, we tested stool samples for their ability to modulate the P-gp/endocannabinoid (homeostasis)-MRP2/HXA_3_ (inflammatory) axis (9, 36). For this analysis, polarized T84 intestinal epithelial cell monolayers were incubated in the presence of stool supernatants, followed by quantification of P-gp and MRP2 protein expression (36, 37). Stool supernatants from AD elders induced a significantly lower expression of functional P-gp than did supernatants from elders with no dementia or other dementia types (Fig. 5A, *P* = 0.017). MRP2 expression was higher in response to stool supernatants from AD elders, but this observation did not reach significance (Fig. 5B).

### Taxonomic predictors of AD can also accurately predict *in vitro* P-gp expression.

**FIG 6:**
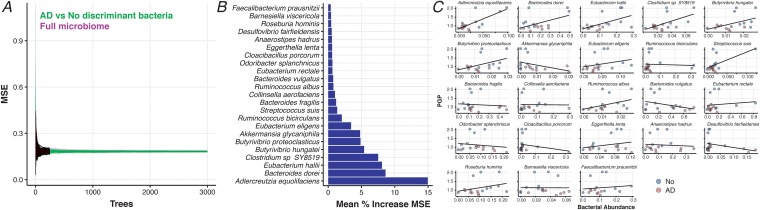
Dysbiotic microbiome species from elders with Alzheimer’s disease can accurately predict *in vitro* P-glycoprotein expression levels. Random forest regression analysis was performed to predict P-glycoprotein expression as a function of the bacterial relative abundances obtained from whole-genome sequencing of elders with Alzheimer’s disease and without any dementia. (A) A side-by-side comparison of the two models’ mean squared error (MSE) as a function of the number of generated trees demonstrates no difference between the MSE of the model obtained by training using only the top 30 features discriminating AD and no dementia (see Fig. 3, green line) and a model obtained by using information of all the available species (purple lines). The regression was performed in both cases starting from 500 different initial random seeds. (B) Variables with positive importance (and hence significantly contributing to the regression) resulted to be only a limited subset of taxa that differentiate Alzheimer’s disease from no-dementia individuals. (C) Species selected by the regression model are predicted to either induce or repress P-glycoprotein expression levels. Observations are colored according to dementia status (AD, red; no, blue).

We next sought to determine if taxonomic predictors of AD could also predict the P-gp response. Applying an approach we have used to decouple the effect of a consortium of gut bacteria on an anti-inflammatory phenotype (38), we built random forest regression models in which we predicted the experimentally measured P-gp expression as a function of the relative bacterial abundances. Our model selected only the top 30 important species for AD prediction as covariates; the other model considered all species profiled in the elder microbiomes (see Fig. 3B). Comparison mean squared error (MSE) as a function of the number of generated trees reveals that there is no difference in MSE between the two models (Fig. 6A), indicating that AD-predicting taxa are sufficient to predict the microbiome-dependent effect on P-gp function. A limited subset of taxa significantly contribute to the regression in both models and thus are likely to be major drivers of loss of intestinal homeostasis (Fig. 6B and C). As seen in our analysis of elder samples, several of the taxa that predict the microbiome-dependent effect on P-gp function are butyrate-producing organisms, which are higher in proportion among the no-dementia patients that induce higher P-gp expression. We then examined the metabolic pathways involved and noted a similar pattern, identifying five of the butyrate-coding enzyme genes that also significantly positively correlate with the measured P-gp induction (Fig. S2).

10.1128/mBio.00632-19.2 FIG S2 Butyrate-coding genes that significantly positively correlate with the measured P-glycoprotein induction. We used Spearman’s correlation to compare the relative abundances of butyrate enzymes, identified from the mixed-effect modeling used to compare Alzheimer’s disease elders to those without dementia, with P-glycoprotein expression. Download FIG S2, PDF file, 0.04 MB. Copyright © 2019 Haran et al. 2019 Haran et al. This content is distributed under the terms of the Creative Commons Attribution 4.0 International license.

## DISCUSSION

Our results advance an understanding of how the intestinal microbiome affects the gut-brain axis in the context of AD. Species-level differences were explored between elders with AD, no dementia, and other dementia types and were combined with key clinical variables including frailty, malnutrition, and medication exposures common among the elderly and known to influence the microbiome composition. This study identified a dysbiotic pattern seen among AD elders in comparison to those without dementia or with other dementia types. This pattern is composed of reductions in key butyrate-producing anti-inflammatory species with increases in species known to have associations with either neurological disorders via inflammation or to other colonic inflammatory states.

AD elders were characterized by lower proportions of key butyrate-producing species, such as members of the Butyrivibrio (*B. hungatei* and B. proteoclasticus) and Eubacterium (*E. eligens*, E. hallii, and E. rectale) genera, as well as species Clostridium sp. SY8519, *R. hominis*, and *F. prausnitzii*. AD elders also had diminished butyrate enzyme-encoding genes than did elders without dementia. Butyrate is an essential metabolite in the human colon. It is the preferred energy source for the colonic epithelial cells, and it contributes to the gut barrier maintenance; also, it has both immunomodulatory and anti-inflammatory properties (39). We infer that lower proportions of butyrate-producing species would lead to a proinflammatory colonic epithelial state.

Elders with AD had increased proportions of bacterial species identified in our analysis that have previously been shown to associate with AD. These include *O. splanchnicus*, a bacterial species with genes that have been associated with the Alzheimer’s pathway (25) and the relative abundance of Odoribacter spp. that has been shown to be increased in transgenic AD mice (40). Additionally, *O. splanchnicus* has also been previously linked to other neurological disorders, specifically autism (41). The human pathogens K. pneumoniae, B. fragilis, and *E. lenta* all have previous known associations to AD and were seen in increased relative abundances in our AD elders. Klebsiella spp. are commensal bacteria capable of assembling extracellular amyloids, the release of which can induce cytotoxicity similar to pathological Aβ in AD patients (42). B. fragilis has known AD associations through its production of lipopolysaccharide (43), and *E. lenta* is a significant human pathogen that is often associated with serious gastric pathology (44). Our model also robustly identified a representative species of the sulfate-reducing Desulfovibrio genus (D. fairfieldensis) to be a high-importance predictor that is enriched in the AD cohort. An increased proportion of sulfate-reducing bacteria has been observed in multiple dysbiotic and colitogenic states (45, 46). Other species with decreased proportions have been seen in other diseases, such as *F. prausnitzii* with Parkinson’s disease (47) and *R. hominis* in patients with Crohn’s disease and ulcerative colitis (48, 49).

Uniquely, our study goes on to demonstrate that stool samples from elders with AD can induce lower P-gp expression levels than seen with samples from elders with either no dementia or other types of dementia. A loss of P-gp expression or a reduction in its function correlates with inflammation in the gastrointestinal tract in mice and humans (9). Reciprocally, clinical evidence indicates that the MRP2 pathway is activated in chronic intestinal inflammation (36). In fact, we are able to demonstrate that the taxa that differentiate the AD microbiome of elders from those without dementia can also predict P-gp expression levels in both of these groups. The top species identified here in predicting P-gp expression include, once again, key butyrate producers, such as members of the *Eubacterium, Clostridium*, and *Butyrivibrio* genera, as well as key butyrate-encoding enzyme pathways. Other species, such as Bacteroides dorei and Akkermansia glycaniphila, have been associated with gut inflammation and autoimmune diabetes (28), and Adlercreutzia equolifaciens, a beneficial microbiota member known to reduce epithelium inflammation (30, 31). Taken together, the microbial taxon members found to best predict the observed P-gp expression are all known to influence colonic inflammation in other pathological states.

In summary, we demonstrate that the microbiome patterns among elders with AD are similar, represented by lower relative abundances of butyrate-producing species and higher relative abundances of taxa known to cause proinflammatory states compared to those with either no dementia or other dementia types. Importantly, this work is an important advance to bridge previous microbiome association studies with AD toward causality by showing how the AD microbiome observed can potentially adversely affect intestinal epithelial homeostasis via dysregulation of the P-gp pathway. Our study supports the conclusion that the relationship between the intestinal microbiome and an altered epithelial homeostasis is a means by which the microbiome impacts this devastating neurodegenerative disorder.

## MATERIALS AND METHODS

### Study setting and population.

This prospective cohort study was approved by the institutional review board at the University of Massachusetts Medical School. This cohort is of NH elders ≥65 years of age who lived in one of four NH facilities located in central Massachusetts. We approached elders across all sites who had been living at that facility for ≥1 month and did not have any diarrheal illness or antimicrobial exposure within the preceding 4 weeks. No elders suffered from dysphagia or had a feeding tube. Any elders with antimicrobial exposure or a diarrheal illness during the conduct of the study were excluded from this analysis.

### Data collection.

We conducted baseline and end-of-study medical record abstraction for factors associated with key study outcomes. These factors included, but were not limited to, age, nutritional status, comorbidities, use of proton pump inhibitors, and frailty (50). Determination of the diagnosis of Alzheimer’s disease dementia or other dementia was made by querying the facility medical record and confirmed by the facility treating physician. Dementia severity was determined by the study staff using the clinical dementia rating (CDR) scoring system. The CDR is a widely used semiobjective instrument for staging dementia severity (51, 52) that has been previously used in reporting gut-brain axis associations among Alzheimer’s disease patients (4). Elders with a CDR score of 0 or 0.5 were categorized as no dementia, 1 as mild dementia, and 2 or 3 as moderate/severe dementia. We obtained age, sex, and medical history from the NH record. Both daily and as-needed medications were obtained from the facility’s medical record. Polypharmacy was defined using the most commonly reported definition of five or more daily medications (53). Polypharmacy has been shown to represent a determinant of gut microbiota composition independent of specific drug classes and has detrimental clinical consequences (54). We categorized elders based off the continuous age variable into 4 age categories for analysis, as follows: category 1, 65 to 74 years; category 2, 75 to 84 years; category 3, 85 to 94 years; and category 4, ≥95 years of age. Frailty was categorized according to the validated and widely utilized Canadian Study of Health and Aging’s (CSHA) 7-point Clinical Frailty Scale (55). This has been previously validated in demonstrating signatures of frailty in the gut microbiota (19, 56, 57). We assessed nutritional status using the Mini Nutritional Assessment (MNA) tool (58–60). Elders were categorized as normal, at risk, or malnourished based on the MNA survey administered to the elders by trained research staff or the nurse caring for the elder if mentally impaired. All elders were enrolled for a total of 5 months in which we monitored for any changes to their care.

### Sample collection and processing.

We collected stool samples longitudinally once a month for up to 4 months from each elder. DNA was extracted from samples using the PowerMag soil DNA isolation kit on an epMotion 5075 TMX liquid handling workstation, according to manufacturer protocols (Mo Bio Laboratories catalog no. 27100-4-EP). Sequencing libraries were constructed using the Nextera XT DNA library prep kit (Illumina, Inc. catalog no. FC-131-1096) and sequenced on a NextSeq 500 sequencing system as 2 × 150-bp paired-end reads.

### Sequence processing and analysis.

Shotgun metagenomic reads were first trimmed and filtered of host contamination using Trimmomatic (61) and Bowtie2 (62) as part of the KneadData pipeline (https://bitbucket.org/biobakery/kneaddata). Reads were then profiled for microbial species relative abundances by mapping them to a NCBI bacterial genomes k-mer database with Kraken (17) and by reconstructing the resulting relative abundance profile at the species level with Bracken (18). Normalized taxonomic abundances were then used for downstream statistical analysis in R (see below). To determine the abundance of enzymes coding for butyrate production, we mapped host-decontaminated shotgun metagenomic reads to a database of butyrate reference protein sequences (63) using ShortBRED (64).

### Data analysis.

We performed *t*-distributed stochastic neighbor embedding to first determine sample similarity with respect to dementia conditions. Permutational multivariate analysis of variance (PERMANOVA) was performed to evaluate inter- versus intraindividual variability in bacterial proportion. To determine genera with significant differences among groups, we used a beta-regression model with zero inflation to predict genus proportion as a function of clinical covariates, including age, frailty, malnutrition score, medications, and dementia state (no/other/AD). To account for the repeated sampling nature, we used generalized linear mixed models using the R package *glmmTMB*. We reported in our analysis genera with a *P* value associated with the dementia state smaller than 0.05.

For the random forest classification according to dementia status, we selected at random one sample per individual and built 3,000 decision trees. This operation was repeated 30 times and using 100 different random seeds. Bacteria were ranked based on the associated mean decreased accuracy distribution across the 30 trials. Bacteria that were ranked in the top 30 important in at least 90% of the 100 seed iterations were considered discriminatory. For the butyrate enzyme differential abundance analysis, we used linear-mixed effect modeling with elder identification (ID) as a random effect. Spearman’s correlation was used to correlate the abundance of butyrate enzymes with P-gp expression.

### Cell culture.

T84 intestinal epithelial cells at passages 50 to 79 (ATCC) were grown in a 1:1 mixture of Dulbecco’s modified Eagle’s medium (DMEM) and Ham’s F-12 nutrient mixture (Thermo Fisher Scientific) supplemented with 14 mM NaHCO_3_, 15 mM HEPES buffer (pH 7.5), 100 units/ml penicillin-streptomycin, and 5% heat-inactivated fetal bovine serum (FBS). Cells were maintained at 37°C and 5% CO_2_. Monolayers were grown on collagen-coated tissue culture-treated 6-well plates (Costar) and used 6 to 8 days after plating. Prior to incubation with fecal supernatants, cells were serum starved for 1 h in serum-free T84 growth medium.

### Fecal supernatant preparation.

Human fecal supernatants were prepared as previously described (65, 66). Fecal samples freshly voided and stored at −80°C were weighed and resuspended in serum-free growth medium to 0.25 g/ml (wt/vol). Samples were homogenized with gentle vortexing and manual grounding with a sterile pipet tip, followed by centrifugation at 10,000 × g for 15 min. The supernatant was sterile-filtered through a 0.22-μm polyethersulfone (PES) filter and diluted 10-fold in serum-free T84 growth medium before adding to the surface of T84 monolayers. Cells were incubated with fecal supernatants for 12 h at 37°C and 5% CO_2_.

### P-glycoprotein detection by flow cytometry.

Cells were washed with phosphate-buffered saline (PBS) and then lifted with 0.25% trypsin-EDTA (Gibco) for 15 min at 37°C. Cells were washed and set to 0.5E6 cells/ml/sample in cold 1× stain buffer (PBS plus 3% FBS plus 1 mM EDTA). Cells were incubated for 30 min in 100 μl stain buffer containing an antigen-presenting cell (APC) anti-human P-gp UIC2 clone (BioLegend catalog no. 348607), or isotype control APC mouse IgG2a(κ) (BioLegend catalog no. 400221). Cells were washed and resuspended in 4′,6-diamidino-2-phenylindole (DAPI; Thermo) for live/dead differentiation. Cell suspensions were filtered through 40-μm nylon mesh prior to data collection on a MACSQuant10 flow cytometer (Miltenyi Biotec). Data were analyzed using the FlowJo software (Tree Star). The geometric mean of the APC^+^ population was computed for each sample and normalized to an untreated medium control sample.

### MRP2 detection by Western blotting.

Cell monolayers were lysed in 1× lysis buffer (20 mM Tris [pH 7.5], 120 mM NaCl, 1 mM EDTA, 1% Triton X-100, 0.5% sodium deoxycholate, 1× protease inhibitor cocktail [Roche]). Lysates were centrifuged at 12,000 rpm for 5 min at 4°C. Supernatants were normalized for protein concentration, separated by SDS-PAGE gels under reducing conditions, and transferred to nitrocellulose membranes. After 1 h of incubation in PBS-based blocking buffer (Li-Cor), blots were incubated overnight with primary antibodies anti-MRP2 (catalog no. Ab3373; Abcam) at a 1:100 dilution or anti-glyceraldehyde-3-phosphate dehydrogenase (anti-GAPDH; catalog no. MAB374; Millipore) at a 1:40,000 dilution. After washing with PBST (PBS plus 0.1% Tween), membranes were incubated for 1 h in the secondary antibody IRDye 800CW goat anti-mouse IgG (Li-Cor) at a 1:5,000 dilution (MRP2) or 1:40,000 dilution (GAPDH). Membranes were scanned using an Odyssey infrared imaging system (Li-Cor). Densitometry analysis was performed using Image Studio Lite version 5.2. Densitometry values for MRP2 were normalized to internal loading control GAPDH.

To predict *in vitro* P-gp induction from microbial proportions and test that AD versus no-dementia discriminatory microbes are responsible for the observed P-gp profiling, we built a random forest regression model. We again used 3,000 trees and 500 different initial random number seeds. The proportion of bacteria resulting to have positive contribution to the MSE were plotted against the P-gp levels in the corresponding samples. One sample *t* test between for the MSE of the model built with preselecting AD versus no classifying features and a model built using all the microbial taxa were performed to test the null of no difference in prediction accuracy.

### Ethics approval.

This prospective cohort study was approved by the institutional review board (IRB) at the University of Massachusetts Medical School (docket H00010892).

### Data availability.

The data sets and code will be made available to the scientific community for further analysis upon written request to John P. Haran.

10.1128/mBio.00632-19.5 TEXT S1 Supplemental references. Download Text S1, PDF file, 0.1 MB. Copyright © 2019 Haran et al. 2019 Haran et al. This content is distributed under the terms of the Creative Commons Attribution 4.0 International license.
