# Supplementary figures and images for: Alzheimer’s Disease Microbiome Is Associated with Dysregulation of the Anti-Inflammatory P-Glycoprotein Pathway
Source: mBio. 2019 May 7;10(3):e00632-19. doi: 10.1128/mBio.00632-19 (PMC6509190; doi:10.1128/mBio.00632-19)

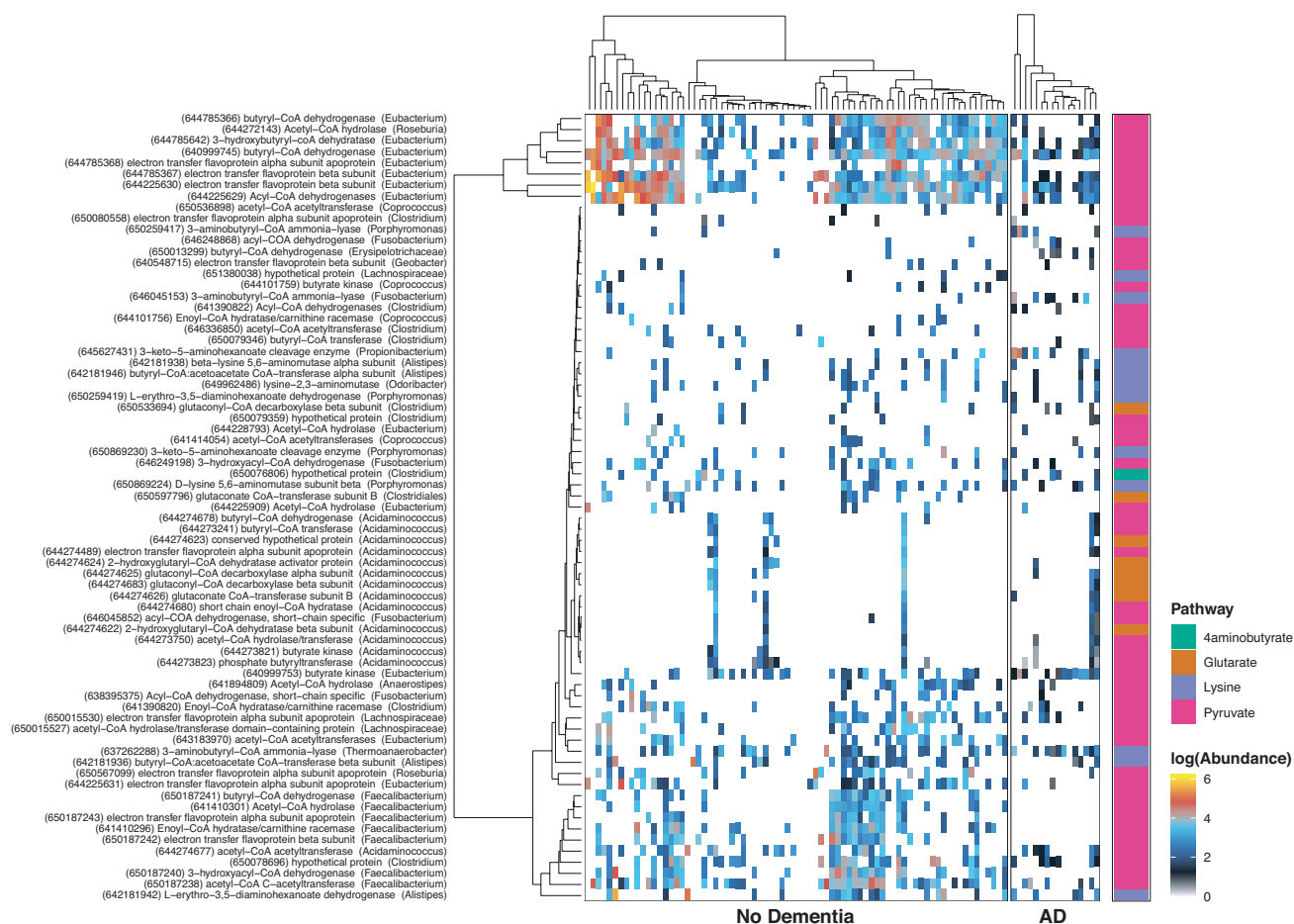

Supplement: FIG S1 [file mBio.00632-19-sf001.pdf]

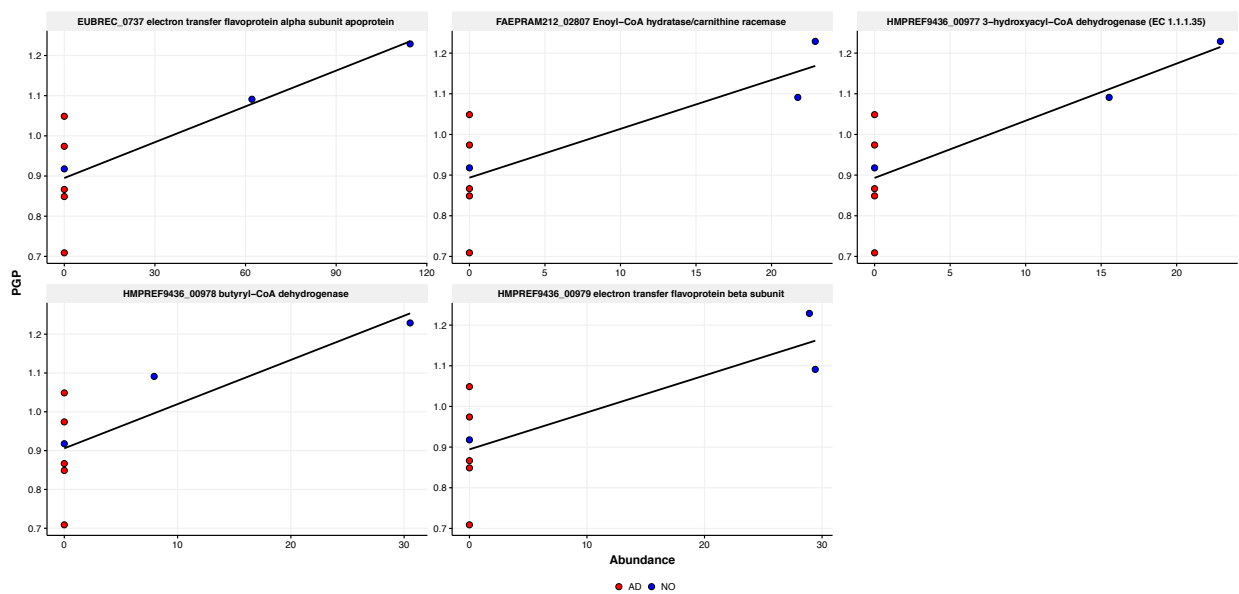

Supplement: FIG S2 [file mBio.00632-19-sf002.pdf]
