# Supplementary material for: Alzheimer’s Disease Microbiome Is Associated with Dysregulation of the Anti-Inflammatory P-Glycoprotein Pathway
Source: mBio. 2019 May 7;10(3):e00632-19. doi: 10.1128/mBio.00632-19 (PMC6509190; doi:10.1128/mBio.00632-19)
Supplement: TABLE S1 [file mBio.00632-19-st001.pdf]

Species	Abundances in AD Compared to No Dementia	Related Conditions	Abundances in Disease Related Conditions	References
**Butyrate Producing Species**				
Anaerostipes hadrus	decreased			[1]
Butyrivibrio hungatei	decreased			[2]
Butyrivibrio proteoclasticus	decreased			[2]
Cloacibacillus porcorum	decreased			[3,4]
Clostridium sp SY8519	decreased			[5]
Eubacterium eligens	decreased			[6]
Eubacterium hallii	decreased			[7]
Eubacterium rectale	decreased			[6]
Faecalibacterium prausnitzii	decreased			[8]
Roseburia hominis	decreased			[9,10]
Ruminococcus bicirculans	decreased			[2]
**Previous Associations with Alzheimer's Disease**				
Bacteroides fragilis	increased		increased	[11]
Eubacterium hallii	decreased		decreased	[12]
Eubacterium rectale	decreased		decreased	[12]
Klebsiella pneumonia	increased		increased	[13-15]
Odoribacter splanchnicus	increased		increased	[16-18]
**Inflammation**				
Bacteroides dorei	increased	Type 1 Diabetes	increased	[19]
Bacteroides vulgatus	increased	Autoimmune Diabetes/Autism	increased	[20-22] [19]
Collinsella aerofaciens	increased	Rheumatoid Arthritis	increased	[23]
Desulfovibrio fairfieldensis	increased	Irritable Bowel Disease/Obesity	increased	[24,25]
Roseburia hominis	decreased	Ulcerative Colitis	decreased	[10]
**Other Neurological Diseases**				
Adlercreutzia equolifaciens	decreased	Multiple Sclerosis	decreased	[26]
Clostridium sp SY8519	decreased	Parkinson's	decreased	[27]
Faecalibacterium prausnitzii	decreased	Parkinson's/ Irritable Bowel Disease	decreased	[10,27,28]

**Pathogens**				
Bacteroides fragilis	increased	Multiple sites	increased	[29]
Desulfovibrio fairfieldensis	increased	Bacteremia	increased	[30]
Eggerthella lenta	increased	Intestinal Infections	increased	[31]
