# Supplementary material for: Alzheimer’s Disease Microbiome Is Associated with Dysregulation of the Anti-Inflammatory P-Glycoprotein Pathway
Source: mBio. 2019 May 7;10(3):e00632-19. doi: 10.1128/mBio.00632-19 (PMC6509190; doi:10.1128/mBio.00632-19)
Supplement: TABLE S2 [file mBio.00632-19-st002.pdf]

Species	Abundances in AD Compared to No Dementia	Related Conditions	Abundances in Disease Related Conditions	References
**Butyrate Producing Species**				
Butyrivibrio hungatei	decreased			[2]
Blautia hansenii	decreased			[32]
Cloacibacillus porcorum	decreased			[3,4]
Clostridium saccharolyticum	decreased			[5]
Eubacterium eligens	decreased			[6]
Faecalibacterium prausnitzii	decreased			[8]
Roseburia hominis	decreased			[9 10]
Ruminococcus bicirculans	decreased			[2]
**Previous Associations with Alzheimer's Disease**				
Bacteroides fragilis	decreased		increased	[11]
Bifidobacterium bifidum	decreased	As a probiotic treatment	increased consumption	[33]
Klebsiella pneumonia	increased		increased	[13-15]
Odoribacter splanchnicus	increased		increased	[16-18]
**Inflammation**				
Akkermansia muciniphila	increased	Type 1 Diabetes	increased	[20]
Bacteroides dorei	increased	Type 1 Diabetes	increased	[19]
Bifidobacterium longum	decreased	Antibiotic Associated Diarrhea	decreased	[34,35]
Collinsella aerofaciens	increased	Rheumatoid Arthritis	increased	[23]
Desulfovibrio fairfieldensis	increased	Irritable Bowel Disease/Obesity	increased	[24,25]
Parabacteroides distasonis	decreased	Multiple Sclerosis	decreased	[36]
Roseburia hominis	decreased	Ulcerative Colitis	decreased	[10]
**Other Neurological Diseases**				
Blautia hansenii	decreased	Autism	decreased	[37]

Faecalibacterium prausnitzii	decreased	Parkinson's/ Irritable Bowel Disease	decreased	[10,27,28]
Parabacteroides distasonis	decreased	Multiple Sclerosis	decreased	[36]
Prevotella denticola	decreased	Parkinson's/ Multiple Sclerosis	decreased	[26,27]
**Pathogens**				
Bacteroides fragilis	decreased	Multiple sites	increased	[29]
Campylobacter jejuni	increased	Gastroenteritis	increased	[38]
Desulfovibrio fairfieldensis	increased	Bacteremia	increased	[30]
Eggerthella lenta	increased	Intestinal Infections	increased	[31]
Ralstonia mannitolilytica	decreased	Nosocomial Infections	increased	[39]
Ralstonia pickettii	decreased	Bacteremia	increased	[40]
