# Supplementary material for: Alzheimer’s Disease Microbiome Is Associated with Dysregulation of the Anti-Inflammatory P-Glycoprotein Pathway
Source: mBio. 2019 May 7;10(3):e00632-19. doi: 10.1128/mBio.00632-19 (PMC6509190; doi:10.1128/mBio.00632-19)
Supplement: TEXT S1 [file mBio.00632-19-s0001.pdf]

## REFERENCES


1. Kant, R., Rasinkangas, P., Satokari, S., Pietilä, T.E. & Palva, A. Genome Sequence of the Butyrate-Producing Anaerobic Bacterium Anaerostipes hadrus PEL 85. *Genome Announc* **3**, e00224-00215 (2015).
2. Duncan, S.H.*, et al.* Wheat bran promotes enrichment within the human colonic microbiota of butyrate-producing bacteria that release ferulic acid. *Environmental microbiology* **18**, 2214-2225 (2016).
3. Levine, U.Y., Looft, T., Allen, H.K. & Stanton, T.B. Butyrate-producing bacteria, including mucin degraders, from the swine intestinal tract. *Appl Environ Microbiol* **79**, 3879-3881 (2013).
4. Looft, T., Levine, U.Y. & Stanton, T.B. Cloacibacillus porcorum sp. nov., a mucin-degrading bacterium from the swine intestinal tract and emended description of the genus Cloacibacillus. *International journal of systematic and evolutionary microbiology* **63**, 1960-1966 (2013).
5. Li, Z., Zhu, H., Zhang, L. & Qin, C. The intestinal microbiome and Alzheimer's disease: A review. *Animal Models and Experimental Medicine* **1**, 180-188 (2018).
6. Eid, H.M.*, et al.* Significance of Microbiota in Obesity and Metabolic Diseases and the Modulatory Potential by Medicinal Plant and Food Ingredients. *Frontiers in pharmacology* **8**, 387 (2017).
7. Engels, C., Ruscheweyh, H.J., Beerenwinkel, N., Lacroix, C. & Schwab, C. The Common Gut Microbe Eubacterium hallii also Contributes to Intestinal Propionate Formation. *Frontiers in microbiology* **7**, 713 (2016).
8. Takahashi, K.*, et al.* Reduced Abundance of Butyrate-Producing Bacteria Species in the Fecal Microbial Community in Crohn's Disease. *Digestion* **93**, 59-65 (2016).

9. Patterson, A.M., *et al.* Human Gut Symbiont Roseburia hominis Promotes and Regulates Innate Immunity. *Frontiers in immunology* **8**, 1166 (2017).

10. Machiels, K., *et al.* A decrease of the butyrate-producing species Roseburia hominis and Faecalibacterium prausnitzii defines dysbiosis in patients with ulcerative colitis. *Gut* **63**, 1275-1283 (2014).

11. Lukiw, W.J. Bacteroides fragilis Lipopolysaccharide and Inflammatory Signaling in Alzheimer's Disease. *Frontiers in microbiology* **7**, 1544 (2016).

12. Cattaneo, A., *et al.* Association of brain amyloidosis with pro-inflammatory gut bacterial taxa and peripheral inflammation markers in cognitively impaired elderly. *Neurobiology of aging* **49**, 60-68 (2017).

13. Shahnawaz, M. & Soto, C. Microcin amyloid fibrils A are reservoir of toxic oligomeric species. *The Journal of biological chemistry* **287**, 11665-11676 (2012).

14. Friedland, R.P. Mechanisms of molecular mimicry involving the microbiota in neurodegeneration. *Journal of Alzheimer's disease : JAD* **45**, 349-362 (2015).

15. Friedland, R.P. & Chapman, M.R. The role of microbial amyloid in neurodegeneration. *PLoS pathogens* **13**, e1006654 (2017).

16. KEGG PATHWAY: Alzheimer disease - Reference pathway for Odoribacter splanchnicus. http://www.genome.jp/keggbin/show_organism?menu_type=pathway_maps&category=Odoribacter splanchnicus&category_type=species.

17. De Angelis, M., Francavilla, R., Piccolo, M., De Giacomo, A. & Gobbetti, M. Autism spectrum disorders and intestinal microbiota. *Gut microbes* **6**, 207-213 (2015).

18. Shen, L., Liu, L. & Ji, H.F. Alzheimer's Disease Histological and Behavioral Manifestations in Transgenic Mice Correlate with Specific Gut Microbiome State. *Journal of Alzheimer's disease : JAD* **56**, 385-390 (2017).

19. Davis-Richardson, A.G.*, et al.* Bacteroides dorei dominates gut microbiome prior to autoimmunity in Finnish children at high risk for type 1 diabetes. *Frontiers in microbiology* **5**, 678 (2014).

20. Aaron, L. The anti-neo-epitopes tissue and microbial transglutaminases are new reliable serological markers in celiac disease diagnosis. *Journal of Clinical & Cellular Immunology* **08**(2017).

21. Finegold, S.M.*, et al.* Gastrointestinal microflora studies in late-onset autism. *Clinical infectious diseases : an official publication of the Infectious Diseases Society of America* **35**, S6-S16 (2002).

22. Bercik, P.*, et al.* The anxiolytic effect of Bifidobacterium longum NCC3001 involves vagal pathways for gut-brain communication. *Neurogastroenterology and motility : the official journal of the European Gastrointestinal Motility Society* **23**, 1132-1139 (2011).

23. Horta-Baas, G.*, et al.* Intestinal Dysbiosis and Rheumatoid Arthritis: A Link between Gut Microbiota and the Pathogenesis of Rheumatoid Arthritis. *Journal of immunology research* **2017**, 4835189 (2017).

24. Xiao, S.*, et al.* A gut microbiota-targeted dietary intervention for amelioration of chronic inflammation underlying metabolic syndrome. *FEMS microbiology ecology* **87**, 357-367 (2014).

25. Rooks, M.G.*, et al.* Gut microbiome composition and function in experimental colitis during active disease and treatment-induced remission. *The ISME journal* **8**, 1403-1417 (2014).

26. Chen, J., *et al.* Multiple sclerosis patients have a distinct gut microbiota compared to healthy controls. *Scientific reports* **6**, 28484 (2016).

27. Houser, M.C. & Tansey, M.G. The gut-brain axis: is intestinal inflammation a silent driver of Parkinson's disease pathogenesis? *NPJ Parkinson's disease* **3**, 3 (2017).

28. Gevers, D., *et al.* The treatment-naive microbiome in new-onset Crohn's disease. *Cell host & microbe* **15**, 382-392 (2014).

29. Wexler, H.M. Bacteroides: the good, the bad, and the nitty-gritty. *Clinical microbiology reviews* **20**, 593-621 (2007).

30. Goldstein, E.J.C., Citron, D.M., Peraino, V.A. & Cross, S.A. Desulfovibrio desulfuricans Bacteremia and Review of Human Desulfovibrio Infections. *Journal of clinical microbiology* **41**, 2752-2754 (2003).

31. Gardiner, B.J., *et al.* Clinical and microbiological characteristics of Eggerthella lenta bacteremia. *Journal of clinical microbiology* **53**, 626-635 (2015).

32. Liu, C., Finegold, S.M., Song, Y. & Lawson, P.A. Reclassification of Clostridium coccoides, Ruminococcus hansenii, Ruminococcus hydrogenotrophicus, Ruminococcus luti, Ruminococcus productus and Ruminococcus schinkii as Blautia coccoides gen. nov., comb. nov., Blautia hansenii comb. nov., Blautia hydrogenotrophica comb. nov., Blautia luti comb. nov., Blautia producta comb. nov., Blautia schinkii comb. nov. and description of Blautia wexlerae sp. nov., isolated from human faeces. *International journal of systematic and evolutionary microbiology* **58**, 1896-1902 (2008).

33. Akbari, E., *et al.* Effect of Probiotic Supplementation on Cognitive Function and Metabolic Status in Alzheimer's Disease: A Randomized, Double-Blind and Controlled Trial. *Frontiers in aging neuroscience* **8**, 256 (2016).

34. Zarrati, M., *et al.* Effects of probiotic yogurt on fat distribution and gene expression of proinflammatory factors in peripheral blood mononuclear cells in overweight and obese people with or without weight-loss diet. *Journal of the American College of Nutrition* **33**, 417-425 (2014).

35. Allen, S.J., *et al.* Lactobacilli and bifidobacteria in the prevention of antibiotic-associated diarrhoea and Clostridium difficile diarrhoea in older inpatients (PLACIDE): a randomised, double-blind, placebo-controlled, multicentre trial. *The Lancet* (2013).

36. Shahi, S.K., Freedman, S.N. & Mangalam, A.K. Gut microbiome in multiple sclerosis: The players involved and the roles they play. *Gut microbes* **8**, 607-615 (2017).

37. Inoue, R., *et al.* A preliminary investigation on the relationship between gut microbiota and gene expressions in peripheral mononuclear cells of infants with autism spectrum disorders. *Bioscience, biotechnology, and biochemistry* **80**, 2450-2458 (2016).

38. Galanis, E. Campylobacter and bacterial gastroenteritis. *CMAJ : Canadian Medical Association journal = journal de l'Association medicale canadienne* **177**, 570-571 (2007).

39. Lim, C.T.S. & Lee, S.E. A rare case of Ralstonia mannitolilytica infection in an end stage renal patient on maintenance dialysis during municipal water contamination. *Pakistan journal of medical sciences* **33**, 1047-1049 (2017).

40. Ryan, M.P., Pembroke, J.T. & Adley, C.C. Ralstonia pickettii: a persistent gram-negative nosocomial infectious organism. *The Journal of hospital infection* **62**, 278-284 (2006).
